# Perspective-Taking With Deictic Motion Verbs in Spanish: What We Learn About Semantics and the Lexicon From Heritage Child Speakers and Adults

**DOI:** 10.3389/fpsyg.2021.611228

**Published:** 2021-03-31

**Authors:** Michele Goldin, Kristen Syrett, Liliana Sanchez

**Affiliations:** ^1^Department of Languages, Literatures and Cultures, University at Albany, SUNY, New York, NY, United States; ^2^Department of Linguistics and Center for Cognitive Science (RuCCS), Rutgers, The State University of New Jersey, New Brunswick, NJ, United States; ^3^Department of Hispanic and Italian Studies, University of Illinois at Chicago, Chicago, IL, United States

**Keywords:** deictic verbs, perspective, heritage speakers, Spanish, context, semantics, pragmatics

## Abstract

In English, deictic verbs of motion, such as *come* can encode the perspective of the speaker, or another individual, such as the addressee or a narrative protagonist, at a salient reference time and location, in the form of an indexical presupposition. By contrast, Spanish has been claimed to have stricter requirements on licensing conditions for *venir* (“to come”), only allowing speaker perspective. An open question is how a bilingual learner acquiring both English and Spanish reconciles these diverging language-specific restrictions. We face this question head on by investigating narrative productions of young Spanish-English bilingual heritage speakers of Spanish, in comparison to English monolingual and Spanish dominant adults and children. We find that the young heritage speakers produce *venir* in linguistic contexts where most Spanish adult speakers *do not*, but where English monolingual speakers *do*, and also resemble those of young monolingual Spanish speakers of at least one other Spanish dialect, leading us to generate two mutually-exclusive hypotheses: (a) the encoding of speaker perspective in the young heritage children is cross-linguistically influenced by the more flexible and dominant language (English), resulting in a wider range of productions by these malleable young speakers than the Spanish grammar actually allows, or (b) the young Spanish speakers are exhibiting productions that are in fact licensed in the grammar, but which are pruned away in the adult productions, being supplanted by other forms as the lexicon is enriched. Given independent evidence of the heritage speakers' robust Spanish linguistic competence, we turn to systematically-collected acceptability judgments of three dialectal varieties of monolingual adult Spanish speakers of the distribution of perspective-taking verbs, to assess their competence and adjudicate between (a) and (b). We find that adults *accept venir* in contexts in which they do not *produce* it, leading us to argue that (a) *venir* is *not* obligatorily speaker-oriented in Spanish, as has been claimed, (b) adults may not produce *venir* in these contexts because they instead select more specific motion verbs, and (c) for heritage bilingual children, the more dominant language (English) may support the grammatically licensed but lexically-constrained productions in Spanish.

## Introduction

Speakers routinely provide information about their disposition on entities or situations in the world based on the words that they choose. For example, a speaker who finds a dish appetizing may describe it as “tasty,” or describe a bad movie “interminable.” Such lexical expressions unambiguously inform the hearer about the speaker's *perspective*. More subtle, however, are verbs that encode information about a speaker's perspective on events.

Take, for example, the utterances in (1). The speaker who delivers these utterances is providing the hearer with a clue about whether they are at the party or not: the version in (1a) with *come* seems to indicate that the speaker is at the party (or will be), while the version in (1b) indicates they are not.

(1) a. Marianna is **coming** to the party.
   b. Marianna is **going** to the party.

The speaker need not be physically at the event to use *come*. Consider the context in (2), where the party has not yet occurred, and yet even here, the speaker's use of *come* or *go* indicates to the hearer whether they intend to be at the party or not. The hearer is more likely to infer that the speaker *will* be at the party in (2a) than in (2b). Likewise, if the party is already happening, the listener is likely to infer that the speaker is currently at the party in (2a).

(2) a. Are you **coming** to the party tonight?
   b. Are you **going** to the party tonight?

What's more, the event need not be in the immediate future or in the immediate proximity, as indicated in (3).

(3) You should **come** visit me in Paris when I'm there next summer.

Nor does the speaker need to be at these locations, as long as the motion is toward the subject. For example, in (4), one need not infer that the speaker was in the hospital in (4a); rather the individual expressed by the matrix subject of the sentence clearly was, and in fact, this version seems more natural than the one in (4b). Thus, in English, *come* can be used to refer to movement between other people and need not be associated with the speaker alone.

(4) a. David told me that Alex **came** to visit him in the hospital.
   b. David told me that Alex **went** to visit him in the hospital.

The examples above illustrate that verbs, such as *come* (and *go*) capture information about motion of a theme (the “figure”) toward or away from a source or goal (the “ground”) (Talmy, 1972). They differ from other motion verbs that also specify a particular direction along a path toward that goal, whose interpretation is invariable, such as *enter*. Instead, their use is contingent upon specific aspects of the context, namely the location of the speech act participants (the speaker, hearer, and referents) relative to a discourse-salient location at a particular place in time (Fillmore, 1966). It is for this reason that they are called *deictic* verbs.

While the precise semantics and pragmatics of *come* and *go* still remain to be worked out, for our purposes here, we adopt a proposal by Sudo (2018), building on previous work by Fillmore (1967) and Oshima (2006a,b). We begin by assuming that when these perspective taking verbs are used, the source is distinct from the goal, and that these verbs impose restrictions on the goal in the form of presuppositions. Oshima (2006a,b) has specifically argued that a speaker's use of *come* requires perspective-taking, whereby they implicitly adopt the perspective of an *anchor* (Levinson, 2003; Roberts, 2014). Since this component of the verb's meaning is presupposed and not assertoric, it is not-at-issue and is projective. Building on this, Sudo has further proposed *come* has an *indexical presupposition* (much like a pronoun or temporal expression does) that the goal of the theme is the location of an individual (which may be the speaker) at the utterance time or reference time. *Go*, instead, does not carry this presupposition, and competes with *come* and triggers an anti-presupposition. When *come can* be used, it *should* be, because of the principle of “maximize presupposition” (Heim, 1991).

Early developmental studies with monolingual English-speaking children demonstrate that children are sensitive to these aspects of meaning for deictic verbs. While data from comprehension tasks seem to indicate that they understand the meaning of *come* before *go*, making errors with the latter even as late as 6 years of age (Clark and Garnica, 1974), less cognitively taxing production studies with younger children (which do not require the child to adopt another's perspective) neutralize this distinction (Richards, 1976). However, both types of studies reveal that with *come*, children allow for either the speaker or addressee to be the reference point (i.e., the goal of the motion) (Clark and Garnica, 1974; Richards, 1976). Young children are also more accurate at recalling events in a fictional story if the description using *come* or *go* by the narrator is consistent with the protagonist's perspective (Rall and Harris, 2000). Even at 2 years of age, samples of spontaneous speech suggest that children produce *come* and *go* in adult-like ways, producing little to no errors of commission, and distinguishing between these and the verb particles with which they combine in expressing orientation toward a goal (Macrae, 1975). Later, by 8–9 years of age, English-speaking children not only recognize the conditions under which these verbs can be used, but also do not require the perspectival anchor to be rigidly defined as the *speaker* (Clark and Garnica, 1974).

Interestingly, this flexibility with the use of *come* appears not to hold cross-linguistically. In Spanish, for example, *venir* (“to come”) has been claimed to only be licensed when the speaker is describing motion directed toward *themselves* (Fillmore, 1967; Lewandowski, 2007). As in English, sentences, such as those in (2) are possible in Spanish, as shown in (5). And just as in English, (5a) is marginal if the speaker is not involved in the organization of the party or not planning to attend. A Spanish version of (3) is also acceptable, since the speaker will be at the reference point.


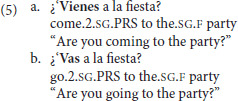


However, Spanish and English have been claimed to diverge when it comes to examples like (4a). Given a context where the speaker is *not* in the hospital at the time of the utterance, then *venir* is not licensed, as shown in (6). Thus, English and Spanish differ on whose perspective can be taken, with Spanish having stricter requirements about this being the speaker.


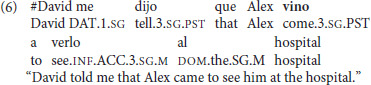


In English, *come* requires that the speaker, addressee, or another discourse-salient individual be at the goal at the utterance or event time (Oshima, 2006a), whereas *go* only references the utterance time (Oshima, 2006b; Sudo, 2018). In Spanish, *venir* (“to come”) seems to only adopt the speaker's perspective at either the utterance or the reference time and cannot express movement toward the *addressee*'s location. *Ir* (“to go”) differs from *venir* in that it denotes movement toward a goal and away from e.g., the speaker (Richardson, 1996; Lewandowski, 2007). These contrasts between perspectival restrictions on the use of *come* are observed not only in English and Spanish, but across a range of the world's languages, as illustrated by Gathercole (1978), Wilkins and Hill (1995), Lewandowski (2007), Nakazawa (2007, 2009), and the numerous references cited in these papers.

This cross-linguistic contrast in the conditions of usage of deictic verbs raises a question about speakers acquiring languages that impose different restrictions on such verbs (as English and Spanish do), especially when one language is more dominant: do these speakers keep the conditions of usage distinct from language to language, or is there cross-linguistic influence of the more dominant language? Previous research with heritage speakers has shown that linguistic skills in the first language are often affected at varying degrees due to intense exposure to a majority language (Dorian, 1981; Lipski, 1993, 1996; Silva-Corvalán, 1994). As Montrul (2004) documents, among the most vulnerable areas of the heritage language to succumb to the second or more dominant language, are the lexicon (Kohnert et al., 1999; Kohnert and Bates, 2002; Köpke, 2002), and “areas where syntax interfaces with other cognitive or extragrammatical areas, such as lexical-semantics, syntax-semantics and discourse-pragmatics” (p. 126, see references therein, and see also Serratrice et al., 2004).

Evidence from adults seems to indicate that the more flexible language influences the other. In Catalan, *venir* allows for the speaker or the addressee to be the reference, while European Spanish carries a stricter requirement that it can only be the speaker. Vann (1998) has shown in an elicited production task that for adult bilingual speakers in Barcelona, there is transfer of the Catalan patterns into Spanish. Likewise, judgment data reported in Chui (2016) shows that while adult native speakers of Spanish require the path of motion to be directed toward the speaker, heritage Spanish speakers (native speakers of both English and Spanish) allow for the hearer to fill this role, ostensibly indicating that these bilinguals may have more flexible parameters in Spanish^1^.

In this paper, we tackle this question of cross-linguistic differences in perspective taking and the implications for dual language learners head-on for the first time in language development by investigating how Spanish heritage bilingual children (for whom the more flexible language, English, is the more dominant language) use Spanish *venir* and English *come* when narrating fictional events in which they themselves are not a participant, but where motion is directed toward the central protagonist in a story (and therefore not the speaker). The prediction based on the current theoretical claims is that in these instances, *venir* would not be licensed in Spanish, but *come* would be in English, and production patterns might reflect this difference. However, given the dominance of English and the context-sensitivity encoded in the lexical semantics of deictic verbs, we might predict that we will observe more “English-like” use of *venir* in these heritage speakers. To quantify the production of *venir*, we compare these heritage speakers to Spanish monolingual speakers from Mexico and Spain and English monolingual speakers from the U.S. (children and adults) narrating in the same task. This strategy allows us to not only track the absolute frequency of *venir* in these non-speaker contexts, but also to compare the relative frequency against the backdrop of other verbs produced in the narrative that more precisely detail the movement in each scene.

Our results reveal that *venir* is produced in narrative in Spanish, patterning similarly to how it is used in English, but only by the young heritage speakers (to the greatest extent) and the children from Spain. One might then argue that the more flexible usage of *come* in the dominant language (English) influences the usage of *venir* in the non-dominant language (Spanish). The heritage children's narratives, however, do not exhibit other signs of crosslinguistic evidence and are quite comparable to those of the monolingual children with respect to accuracy in using compound time referencing and embedded structures (Pearson, 2002). This fact, and the productions by monolingual children in Spain with no exposure to English, led us to follow up with a judgment study with Spanish speaking adults from Mexico and Spain, as well as bilingual Caribbean speakers of Spanish who represent the input of the heritage children, to further investigate whether perspective shift with the use of *venir* is in fact viable in Spanish, contrary to the claims in the theoretical literature.

The results of the judgment study indicate that while Spanish speaking adults do not actually *produce venir* in a narrative, they are actually willing to *accept* it in highly similar contexts when the motion is directed toward the protagonist, and even more so if the word order constraints driven by the unaccusative status of the verb are satisfied. As *venir* is an unaccusative verb, Spanish prompts the need for this very to appear in VS word order in focus-neutral sentences (Burzio, 1986).

These findings therefore indicate that previous theoretical claims about deictic verbs in Spanish should be revisited, since they do not appear to be as strict as previously assumed. Given that this use of *venir* is not observed across *all* children, we propose that variation across groups could be attributed to differences in the restrictions the heritage children, as well as one monolingual group of children, impose on deictic verbs. See Appendix A for a list of the full range of verbs children produced in the corpus study).

Additionally, in the case of the heritage children group, perspective shift could be favored because in the heritage speaker, increased activation of particular features in the more dominant language and the language with more flexible parameters (Putnam and Sánchez, 2013) exaggerates a context-driven, perspective-taking use of *venir* that Spanish does in fact allow. This position is supported by previous research showing that areas that lie outside core aspects of syntax, which are inherently unstable because of their dependence on the context, are most susceptible to cross-linguistic influence (Montrul, 2004; Serratrice et al., 2004).

## Corpus Study

### Corpora and Speakers

The data for analysis were extracted from five corpora in the “Frog Story Corpora” section of the CHILDES database (MacWhinney, 2000), all reporting data from the same story, the wordless picture book *Frog where are you?* by Mercer Mayer (1969). Our primary corpus included the productions of 80 heritage Spanish-English bilingual children, reported in Pearson (2002). These children were living in Miami, Florida, all born in the United States and English dominant, and recruited from 5th grade classes (age 10–11) in Miami-Dade County Public Schools. They narrated 1 day in English, and another day in Spanish, approximately a week after the first language was recorded. The order of the language being elicited was counterbalanced. The bilingual children were compared with a group of 40 monolingual speakers of English living in the same county, also reported in Pearson (2002). These English monolingual children were from households where only one language was spoken. Further demographic information is reported in Pearson (2002).

We then further identified comparison groups of child and adult monolingual speakers who do not live in language contact environments and therefore do not experience lexical competition with English as the bilingual speakers did. The first group, from Aguilar (2015), contains production from 12 year old (*n* = 10, mean age = 12;2) and 6 year old (*n* = 10, mean age = 6;8) monolingual Spanish speaking children living in Mexico. The second group, from Sebastián (1991) and López Ornat and del Castillo (1994), contains productions from monolingual 8 and 9 year old Spanish speaking children living in Spain (López-Ornat: *n* = 20, mean age = 8;0; Sebastián: *n* = 12, mean age = 9;6). The final two groups contain productions from 12 monolingual Spanish speaking adults in Spain (Sebastián, 1991) and 12 monolingual English adults in the United States (Berman and Slobin, 1994), each group ~20 years of age. Thus, in total, we compared four sets of monolingual children and two sets of monolingual adults of varying dialects to our target group of Spanish heritage bilingual children.

Each speaker participated in the same narration task. Following the protocol outlined by Berman and Slobin (1994), the participants first looked through the picture book once from beginning to end, and then narrated it on their own, turning the pages at their own pace. The narrations were then transcribed, and shared via the CHILDES database (MacWhinney, 2000). It is these transcriptions that we analyzed for each corpus, using the same coding schema.

### Coding

Because the task involves narration of a story, rather than a speaker-hearer interaction, this task affords us a prime opportunity to not only evaluate the participants' willingness to produce *come* or *venir* while adopting a non-speaker (and non-addressee) perspective, but also to quantify over and compare instances of such productions across participant groups. Let us recall that previous literature has described that, in Spanish, *venir* is only licensed when the speaker is describing motion directed toward themselves (Fillmore, 1967; Lewandowski, 2007), whereas in English come can more flexibly be used to denote motion toward either a speaker, addressee or any other discourse-salient individual (Oshima, 2006a).

We targeted six main scenes from the story when characters come onto the scene, in a motion directed toward the non-speaker protagonist, as summarized in (a)–(f) below. However, in the case of (a), the frog does not come, and in the case of (f), the frog family is encountered from the boy's perspective.

(a) the boy who has lost the **frog** calls to him to come back
(b) a **mole** peeks out of a hole in the ground and bites the boy's nose
(c) a swarm of **bees** emerges from a hive and chases the boy's dog
(d) an **owl** swoops down from a tree and frightens the boy
(e) a **deer** (which has blended in with the tree branches) rises up from behind a rock, with the boy clinging to its antlers
(f) the boy discovers his frog, which is part of a **frog family**, behind a log

These scenes were then coded for all descriptions of the salient agent (bolded above). All of the transcripts were coded by the first author, a native bilingual speaker of both English and Spanish, in consultation with the other two authors. For each relevant production, the main verb was recorded. These instances were then tabulated in total for each scene, and then for each participant group.

### Predictions

Given the cross-linguistic differences between English and Spanish in the licensing conditions of *come/venir* (“to come”), and the independently-observed instances of language transfer attested in adult heritage and bilingual speakers from a dominant language that is less restrictive to a more restrictive one^2^, we generated the following predictions.

First, we predicted that monolingual Spanish speaking adults and children would not produce *venir* in the relevant scenes in their narrations and would instead opt for other verbal alternatives that (perhaps more precisely) described the movement of the scenes, while the monolingual English children and adults would allow such uses, as long as the lexical and felicity conditions were appropriately satisfied (i.e., motion was toward the protagonist). Second, given the context-sensitivity of this lexical item, we predicted that production of this lexical item would vary from scene to scene, depending on the type and direction of motion. Third, for the heritage speakers, we generated two different predictions. Given the dominance of English for them, we predicted they might pattern with the monolingual English speakers in English, but also that this dominant, more flexible language could influence the non-dominant, more restrictive language, such that the heritage speakers would produce *venir* in Spanish in a way that more closely resembled their English. Now, we also leave open a final possibility that the production of *venir* across the targeted groups might reveal something unexpected relative to theoretical predications, which were not based on systematically controlled data.

### Results

The results from the narration study are summarized in Table 1. We begin by looking at the production of *to come* and *venir*.

**Table 1:** Raw count and percentage of productions of “to come” (as *come* or *venir*) in six scenes and overall, compared to other verbs.

	**Verb Production**					
**English**	***to come***		**Other Vs**		**Total Vs**	
English adults	1	2.2%	36	78.3%	46	100%
English 10/11 year olds	10	5.8%	115	67.3%	171	100%
Bilingual heritage 10–11 year-olds	14	0.04%	223	63.4%	352	100%
**Spanish**	***venir***		**Other Vs**		**Total Vs**	
Spanish adults	0	0.0%	26	52.0%	50	100%
Spain 8/9 year olds	5	3.4%	71	48.6%	146	100%
Mexico 6 year olds	0	0.0%	31	60.8%	51	100%
Mexico 12 year olds	0	0.0%	80	83.3%	96	100%
Bilingual heritage 10–11 year olds	12	4.0%	196	64.7%	303	100%

Note that what matters is not necessarily the *absolute* number of hits of *venir*, but rather the rate of production *relative* to 0% and to other verbs in the target language, because previous literature predicts that productions of *to come/venir* are licensed in narration in English, but not in Spanish. What stands out is that the Spanish heritage speakers are producing *venir* at a rate comparable to their production of *come* in English, and comparable to the production of English monolingual children's production of *come*. By contrast, monolingual Spanish-speaking children of the same age from Mexico, and monolingual Spanish adults from Spain *never* produce *venir* in these contexts.

Recall that *come* is not the only deictic verb; it has a counterpart, *go* (*ir*), which is subject to similar contextual restrictions. This verb was produced in the “bee” scene, and exhibited striking similarity to *come/venir* in the frequency of its production: as with *venir*, the heritage speakers patterned with the monolingual English speakers and the Spanish monolingual children from Spain in their production of *ir*. In fact, the bilingual speakers seem to produce *ir* more frequently than all other groups, a pattern for which we do not have an explanation, but keep in mind this is one verb and one scene. For both deictic verbs, no Spanish monolingual adults ever produced them in their narratives (see Table 2).

**Table 2:** Raw count and percentage of productions of “to go” (in Spanish, *ir*) relative to all verbs in the “bee” scene.

**English**	***go (+PP)***	
English adults	0	0.0%
English 10/11 year olds	4	8.9%
Bilingual heritage 10/11 year olds	14	17.1%
**Spanish**	***ir (+PP)***	
Spanish adults	0	0.0%
Spain 8/9 year olds	5	17.2%
Mexico 6 year olds	0	0.0%
Mexico 12 year olds	0	0.0%
Bilingual heritage 10/11 year olds	15	21.4%

**Figure 1:**
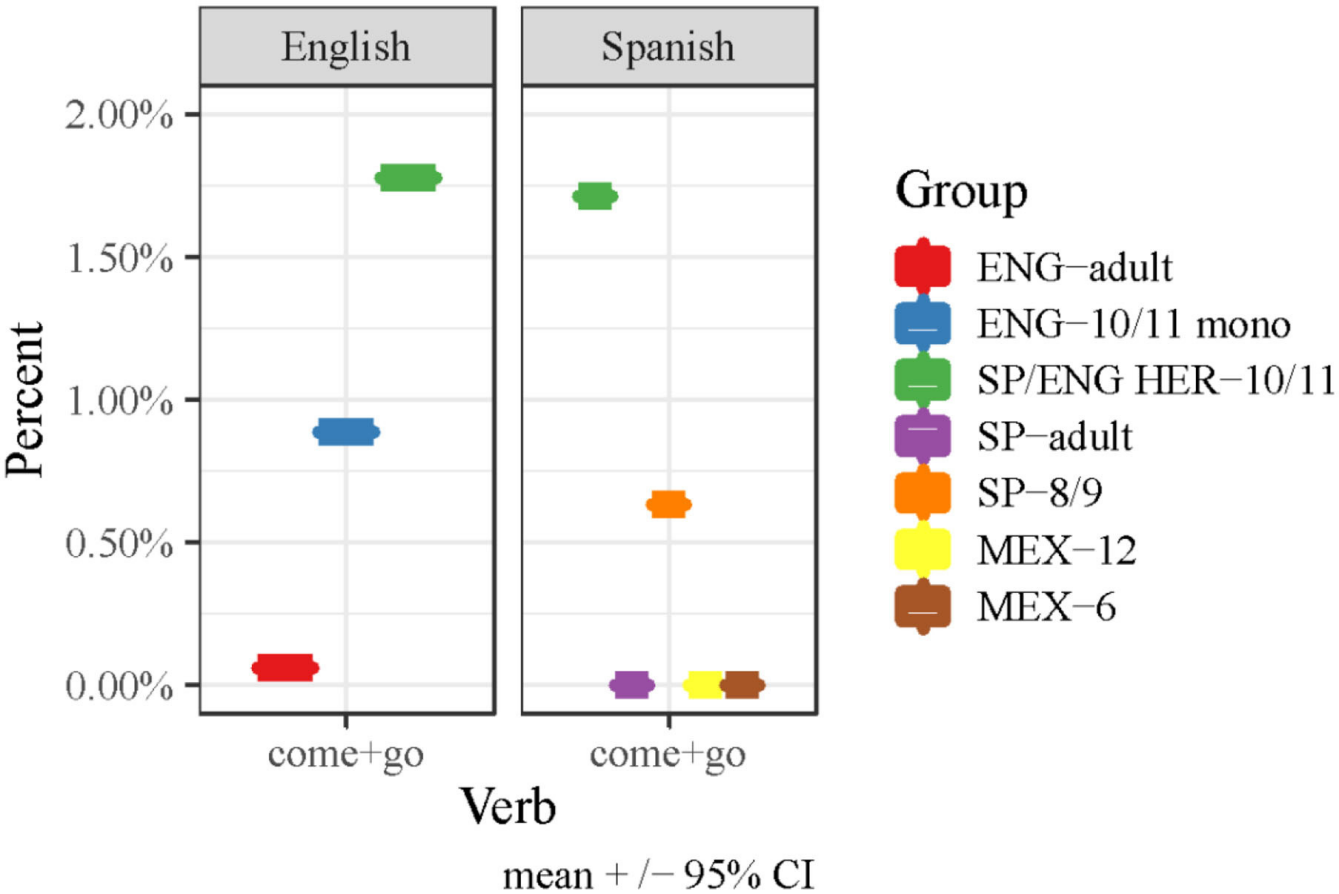
Percentage of use of the verbs “come” (as *come* or *venir*) and “go” (as *go* or *ir*) out of all 1,576 verbs produced in all coded scenes.

To highlight the pattern of combined deictic verb usage by all participant groups across scenes out of all total verbs (*n* = 1,576), in Figure 1, we collapse over both deictic verbs (*come/venir* and *go/ir*) (*n* = 80). Once again highlighted is the increased usage of these verbs by the heritage bilingual children relative to the other groups. The data were analyzed in R version 1.1.5019 (R Development Core Team, 2012) using a GLM to examine the counts of *come* and *go* as a function of group. Given the count nature of the participants' productions, the data were modeled using a Poisson regression with a log linking function. Nested model comparisons were used so as to be able to choose the most parsimonious model that adequately fit the data. The production of *come* and *go* in each group in Spanish was significantly different (*p* < 0.001) (see Figure 1) such that the bilingual heritage children produced more instances of *venir* than the children from Spain and all other groups (who did not produce *venir* at all).

The general trends captured above are borne out in the productions across multiple heritage speakers, examples of which are included in the Appendix B, along with productions from the Spanish monolinguals in the same story context where *venir* was not used.

### Discussion

The results of our corpus study demonstrate that the young heritage speakers in this narration task produced *venir* in a way that was more reminiscent of English than Spanish, considering the felicity conditions of the two languages previously outlined in the theoretical literature. In support of our hypothesis about cross-linguistic influence from English, the monolingual children from Mexico and the monolingual adults from Spain never produced *venir*. However, the handful of productions of venir from the children in Spain led us to revisit our hypothesis and take a closer look at the productions.

Importantly, the heritage speakers are not producing *venir* for wont of a more expanded lexicon in Spanish. Indeed, the comparison of all verbal productions by the five groups of Spanish speakers across each of the target scenes reveals a comparable range of verb choices, which are *contextually conditioned* by the type of motion in the scene, as shown in the tables presented in Appendix A. Relatedly, commenting of the narrative proficiency of the heritage bilingual children, Pearson (2002) herself said of this group, “The bilingual children's stories exhibited age-appropriate skill in difficult tasks like creating a unified plot, motivating events through reference to internal states, and providing narrator's comments on the unfolding story… [they] were accurate in using compound time referencing and embedded structures which distinguished their own thoughts from those of the characters. In these ways, their responses to the complex demands of the story genre were comparable to those of their monolingual peers…” (p. 169). Thus, at both lexical and discourse levels, these children were comparable to other Spanish speakers.

What is also highlighted in these individual productions is that these heritage speakers produce narratives that reflect competence comparable to the Spanish monolingual speakers in the form of word order and argument drop. For example, rather than recount that “a reindeer came” (SV) the heritage children are more likely to say “came a reindeer” (VS), the expected word order with unaccusative verbs like *venir* in focus neutral sentences in Spanish (Burzio, 1986), though there are a few instances of SV productions in these scenes as well. While in English, they produce the subject, in Spanish, they allow for the subject argument to be omitted, just as the monolingual children and adults do and is expected in Spanish (Camacho, 2013). Thus, while they diverge from monolingual speakers at the level of the lexicon (with the exception of the European Spanish children), they converge when it comes to topicalization strategies at the syntax-information structure interface.

At the same time, there were other signs of their bilingual status, such as mismatching gender agreement [as in (7)], missing direct object markers [as in (8)], and English lexical insertions [as in (9) and (10)]^3^.


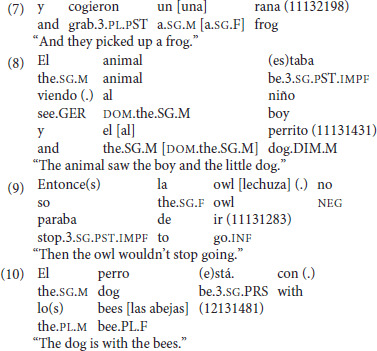


The heritage bilingual children were consistent with all groups of Spanish monolinguals in areas, such as word order for topicalization and argument omission, but their occasional mismatching gender agreement^4^ and inverted word order seemed to reflect the influence of English on their production. Thus, where they can be said to have differed from their monolingual Spanish-speaking peers was in their increased use of perspective-taking verbs and morphosyntactic accuracy. Their narrative skills were otherwise comparable. Influence of English seems to be observable in specific loci (the lexicon and morphology), but not at the interface between syntax and information structure, as evidenced by the presence of subject-verb inversion in their narratives.

However, the Spanish-speaking children from Spain also produced a handful of instances of *venir* and *ir* in the same contexts. One might conjecture, then, that the production of perspective-taking verbs in the target heritage child group is in fact licensed by both English and Spanish (in principle), raising the possibility that some varieties of Spanish may in fact allow for such non-speaker-oriented uses, and perhaps that this pattern is not reflective of cross-linguistic influence *alone*, but rather the influence of English activating an option allowed by the Spanish grammar in some dialects. Thus, though the number of tokens of *venir* produced in this narration corpus study is not necessarily high, it has introduced the question of whether Spanish-speaking adults might *permit* such uses, even if they might not *produce* them. This possibility has not previously been considered, since earlier studies have only assessed production.

To answer this question, we designed an online judgment study, which we administered to adult native speakers of Spanish, targeting the dialects of Spanish spoken in Mexico and Spain, as well as Caribbean and Latin American Spanish, the varieties represented in the input of the bilingual children in the narrative task. Our goal was to investigate the acceptability of non-speaker-oriented *venir* by native speakers, who are presumably providing the language model for the monolingual and bilingual children in this corpus study.

## Acceptability Experiment

### Study

#### Participants

Participants were recruited over social media (Facebook) and on Prolific. A brief demographic questionnaire at the start of each study asked participants to indicate their geographic region of habitation, the place where they were born, the languages they spoke, their first language(s), how much Spanish they used on a daily basis, and their level of education.

One hundred and two adults with Spanish as their first language participated. Participants were divided into three groups: adults from and living in Mexico (MX), adults from and living in Madrid, Spain (SP) and bilinguals of Caribbean and Central American origin living in the U.S. (US). This third group was chosen to reflect the varieties of Spanish available in the input of the bilingual children analyzed in the corpus study. In the MX group, 33 participants were excluded from analysis for not meeting the inclusion criteria including having been born in or living in a country other than Mexico (*N* = 31), and using Spanish <80% of the time on a daily basis (*N* = 3). All remaining participants had some knowledge of English, but used Spanish on a daily basis 80–100% of the time. All had a minimum of high school education, and most (17 of the 20 final participants) had university degrees.

In the SP group (adult speakers from Spain), one participant was excluded from analysis for using English on a regular basis 50% of the time. Of the 21 final participants, 16 had some knowledge of English while the other five knew no other languages. All reported using Spanish 75–100% of the time. All had a minimum of high school education, and 16 had university degrees. In the US group, eight participants were excluded from analysis for not meeting the inclusion criteria including having been born in a country outside Central America or the Caribbean (*n* = 5), living in a country other than the United States (*N* = 1), or having English as a first language (*n* = 2). All remaining participants had knowledge of English and used Spanish on a daily basis 15–100% of the time. All had a minimum of high school education, and most (24 of the 25 final participants) had university degrees. The 25 final participants spoke varieties of Spanish from Cuba (*n* = 5), Puerto Rico (*n* = 8), the Dominican Republic (*n* = 8), Costa Rica (*n* = 1), and Guatemala (*n* = 3).

#### Stimuli and Procedure

The study was implemented and administered over Qualtrics, using a url distributed online. Participants completed two training items before proceeding to the main experimental session. One featured *venir* in a canonically acceptable speaker-addressee situation, and another was an instance of gender agreement mismatch, which was ungrammatical.

There were 35 test sentences, and five filler sentences targeting gender agreement mismatch, pseudorandomized and presented on individual screens with page breaks within each item, for a total of 40 experimental items.

Test stimuli were constructed to elicit judgments of acceptability of motion toward and away from a speaker/hearer or a protagonist in narrative. There were 35 test items with perspective-taking verbs featuring either *venir* or *ir*, divided into seven distinct categories based on context and motion toward/away from entities. All target sentences were preceded by a brief introduction that provided context.

The first category (*n* = 5) featured motion away from a protagonist using ir and the second category (*n* = 5) featured motion away from a protagonist using venir. It was expected that *ir* would be acceptable in this context, as in (11), but that *venir* would be unacceptable, as in the sentence with *venir* in (12) uttered by a speaker not at the restaurant.


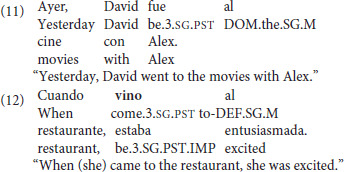


In the third and fourth categories there was motion *toward the hearer* in a speaker-addressee dialog (*ir*: 5; *venir*: 5). All items featuring *ir* were expected to be acceptable, as in (13), but *venir* was expected to be unacceptable (Fillmore, 1967; Oshima, 2006a,b; Lewandowski, 2007; Sudo, 2018) as in the sentence with *venir* in (14).


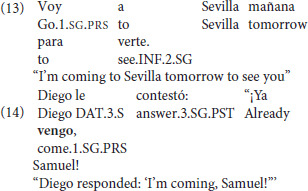


The following three categories depicted motion toward a protagonist using *venir* (i.e., a non-speaker) in a narration context, motivated by the kind produced by the bilingual children in the Frog Stories of the corpus study. The first of these three categories presented *venir* with a null subject, as in (15), the second presented *venir* in VS word order due to its status as an unaccusative verb (Burzio, 1986), as in (16), and the third presented *venir* in SV word order which was expected to be the least acceptable of the three, as in (17), since this word order is non-canonical with an unaccusative verb. These three categories were based on the productions of the bilingual children vis à vis the literature on the constraints on *venir* in Spanish (Gathercole, 1978; Vann, 1998; Lewandowski, 2007). Thus, their acceptance could be revealing of patterns not attested in the previous literature.


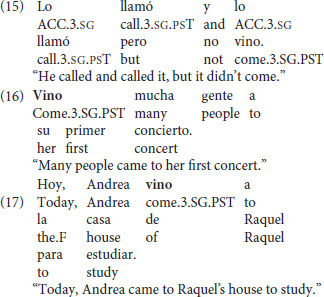


Finally, there were five filler items showing ungrammatical gender and number disagreement that were expected to be unacceptable. For a complete list of test and filler items, see Appendix C.

For each item, paricipants were asked to decide if it sounded good (*suena bien*) or not (*suena raro*) by checking a box, as in (14).


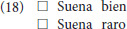


There was also an empty text box accompanying the “raro” option, where participants were allowed to offer an alternative if they so desired, and were instructed to change at most one word. This option allowed us to see if those participants who selected “raro” actually changed the verb or made a change elsewhere (e.g., adverbs, word order, verb tense, etc.).

#### Results

We begin by establishing a baseline. There was 7% acceptability of ungrammatical filler items by the Mexico group (MX), 11% by the Spain group (SP) and 17%^5^ by the US group. As expected, acceptability for the use of *ir* was high when motion was directed Spain from the speaker. The Mexico group accepted items in this category at a rate of 95%, the SP group at a rate of 94% and the US group accepted these at a rate of 98%. Also as expected, acceptability for the use of *venir* was low for this context (MX: 26%; SP: 10%, US: 26%).

The data for the three target categories depicting motion toward the protagonist were analyzed in R version 1.1.5019 (R Development Core Team, 2012) using a GLMM to examine acceptability (0,1) as a function of group (MX, SP, US) and category (*venir* with null subject, *venir* with SV word order, *venir* with SV word order). Given the categorical nature of the participants' responses (acceptable/unacceptable), the data were modeled using GLMMs with a binomial linking function. The predictor “group” was dummy coded with US participants set as the reference level and the predictor “category” with “Motion toward protagonist with null subject” set as the reference level. Main effects and higher order interactions were tested using nested model comparisons.

**Figure 2:**
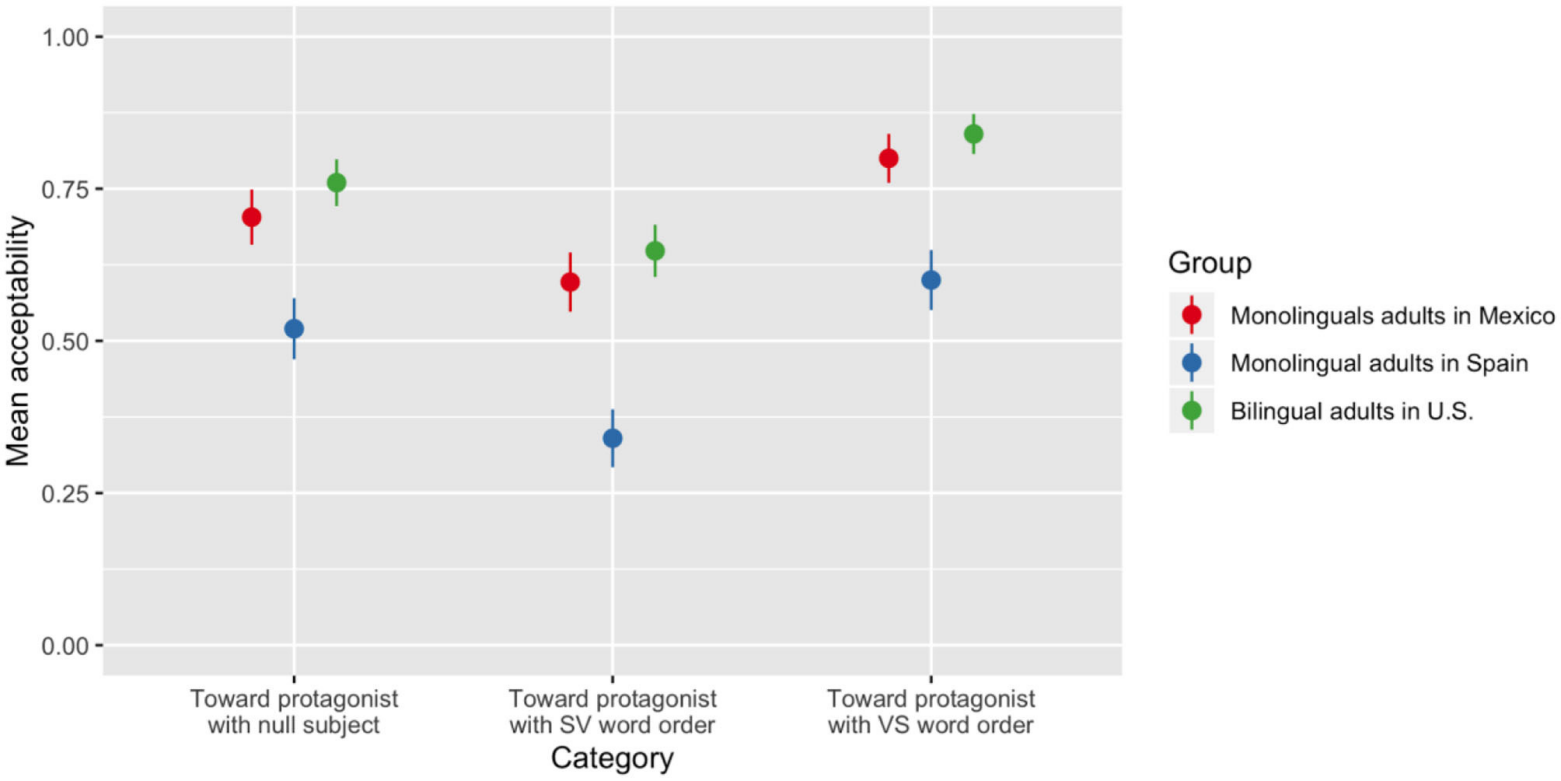
Percentage of acceptability of items in the target categories by group.

The analysis yielded a main effect of group [$\chi_{\left(2\right)}^{2}$ = 14.69, *p* < 0.001]. The SP group exhibited significantly lower acceptability across all three categories than participants in the MX and US groups (β = −1.6; SE = 0.44; z = −3.67; *p* < 0.001). There was also a main effect of category [$\chi_{\left(2\right)}^{2}$ = 48.19, *p* < 0.001] such that items in the “Motion toward protagonist with VS word order” category were accepted at a higher rate than both other categories by all three groups (see Figure 2). There were no interactions.

For these three categories, participants overwhelmingly provided *ir*, or the verbs *aparecer, ver, acercarse*, and *llegar*, as an alternative to *venir* for items they rejected. These were verbs that were also produced in the frog story narratives.

Furthermore, for items in the hearer-speaker dialog categories, participants in all three groups responded as expected. Items presenting *ir* to denote movement toward the hearer were accepted at a rate of 86% by the MX group, 88% by the SP group, and 88% by the US group. However, items using *venir* to signal movement toward the hearer were greatly rejected, with an acceptability rate of 4% from the MX group, 10% for the SP group, and 16% from the US group. In a second GLMM examining acceptability (0,1) for items in the category “*venir* to denote movement toward the hearer” as a function of group (MX, SP, US), there was no effect of group (*p* = 0.99). In other words, acceptability for this kind of use of *venir* was low for all three dialectal groups.

#### Discussion

In this judgment study, Spanish-speaking adults from and in Mexico and Spain and bilingual English-Spanish adult speakers of Caribbean and Central American Spanish living in the U.S. accepted *ir* in speaker-hearer dialog contexts in which motion was speaker-oriented but rejected *venir* to denote this same movement, in line with our predictions based on the previous literature (Oshima, 2006a; Lewandowski, 2007). At the same time, however, there were dialectal differences between the groups. The MX and US groups exhibited high acceptability (well above chance) for all items in which *venir* was used to describe motion toward a non-speaker protagonist in a narrative context. The SP group also accepted these items at a rate higher than originally anticipated, but at a rate significantly lower than the other two groups. All three groups also showed a clear preference for the use of *venir* in VS word order. Recall that VS word order is prompted by unaccusative verbs in focus-neutral sentences (Burzio, 1986). Thus, speakers were not only picking up on the pragmatic-semantic discourse conditions that had to be satisfied for *venir*, but also the word order restrictions prompted by syntax.

The items in the target categories were similar to the types of utterances with *venir* produced by the heritage speakers and the children from Spain in the frog stories. This high percentage is surprising, as it is not predicted by prior theory and if Spanish did not allow this verb to describe anything other than motion toward the speaker, the rating of “Bueno” should be considerably lower, close to zero and comparable to the acceptability rating for the ungrammatical filler items or the use of venir to denote motion toward a hearer in dialog. Based on this pattern, we returned to the productions from the frog stories to evaluate the word order in the narratives in which *venir* was produced. It is important to note that the US group (representing the type of input the heritage bilingual children might have received) exhibited the highest acceptability of items in the target categories and also showed sensitivity to word order. Therefore, it is possible that the bilingual children may have received such utterances in their input.

In sum, the results reveal that Spanish-speaking adults of various dialects show a wider range of acceptability than previous literature claims is possible, and then their production reflects. Based on the acceptability data, it appears that it is possible to shift perspective with *venir* in Spanish, especially when certain discourse conditions are satisfied, adopting the point of view of a protagonist in narrative, but not that of a hearer in dialog. These findings not only shed light on the productions of the young heritage speakers originally analyzed in the frog story narrations, but on the Spanish language as a whole—in particular, the discourse conditions required by the pragmatics and semantics of perspective taking verbs, such as *venir*, as well as the syntactic conditions, that is not documented in the theoretical literature.

## General Discussion and Conclusions

Deictic verbs of motion, such as *come* and *go* present us with an opportunity to investigate what happens when a learner acquires two languages that differ in their contextual requirements on verb use. In our case, the conclusion arising from the theoretical literature is that *come* in English may be used in a wider range of perspectival circumstances in English than *venir* is in Spanish, since the claim is that while English allows for the goal of the theme of *come* to be the location of an individual, such as the speaker or a salient non-speaker, Spanish requires *venir* to describe motion along a path to the *speaker*, and the speaker alone.

In this paper, we asked whether child heritage speakers of Spanish whose dominant language is the more flexible English would pattern more with English or Spanish monolingual peers in their use of these verbs in Spanish, perhaps showing influence of the more flexible, dominant language. Indeed, we observed that their pattern of usage of *venir* in Spanish parallels that of *come* that they produce in English and that is produced by English monolingual speakers, but children from Spain having no contact with English, unexpectedly produced a few similar utterances with *venir*. Specifically, both groups of children produced instances of *venir* that resembled English *come* in that they denoted motion toward a non-speaker (i.e., a protagonist in a narrative), and that the syntactic construction in which they appeared was almost always VS required by *venir* being unaccusative. This indicated that while a conclusion about cross-linguistic influence from English on the heritage speakers is suggestive, it could not be the whole story.

Since the adult monolingual speakers did not produce such instances of *venir* freely, it is clear that the monolingual children from Spain were not obtaining the pattern from the input. Thus, this could not have merely been an instance of overgeneralization on the part of the children that was gradually pruned out in development (as the case is with e.g., the overgeneralization of the past tense morpheme in English, the production of an intransitive verb, such as *giggle* in a transitive frame, or application of word labels for categories like “dog” outside of the basic level), since other monolingual children acquiring another variety of Spanish outside of Spain did not do something similar, and instead demonstrated an adult-like production pattern.

We therefore hypothesize that the monolingual children from Spain were acquiring an aspect of the representation (perspective shift in motion toward protagonist) that *can be* licensed in Spanish (at least in comprehension), which was subsequently pruned out in production. To test this hypothesis, we administered a complementary judgment study to adult Spanish speakers in Mexico, Spain and the United States, and found that the very same types of examples produced by the children were ones that were found to be acceptable by many adults. What then, do we conclude about the young heritage speakers and Spanish as a whole? We think the following picture emerges.

English and Spanish share a common verb: *to come* and *venir*. These verbs overlap in their semantic representation (motion toward a goal), but diverge in their licensing conditions (whether that goal must be the speaker or whether the verb can take on the perspective of a non-speaker, such as a protagonist in a narrative or the addressee). The increased frequency of the more dominant language (English) opens the floodgates for the Spanish verbal correlate to be licensed in children's production more frequently and in a context in which Spanish monolingual adults would not use it (by e.g., analogy or feature activation). In this, the heritage children and the Spanish-English bilingual adults coincide. Monolingual adult Spanish speakers seem to be less likely to *produce venir* in narrative since they have at their disposal a wider set of verbs that more precisely describe the motion they wish to denote. This, of course, does not mean that adults never use *venir* in narrative, but further research is needed to confirm this.

The heritage speakers thus may be experiencing cross-linguistic influence from English, but precisely in a place where pragmatics and the context influence usage. It is important to note that this process may also occur in the Spanish of the bilingual adults providing the input for the heritage children, taking into account that participants in the US group in the judgment study showed high acceptability of English-like non-speaker-oriented uses of *venir*. Given this combined pattern, we argue in line with Putnam and Sánchez (2013), that the increased exposure to the lexical items of the dominant English language and lower frequency of exposure to the corresponding lexical items in Spanish due to reduced input results in higher levels of activation of English and variable strength of the association between the features of the lexical expressions, prompting the learner to license *come* in production from a wider range of perspectives than the less dominant language, Spanish. This increased activation of particular features in the more dominant (and more flexible) language (Putnam and Sánchez, 2013) unlocks a context-driven, perspective-taking use of *venir* that Spanish *does* in fact allow and that more closely approximates the range of possibilities in English.

This position about a specific type of cross-linguistic influence is supported by independent claims that the aspects of language that are most susceptible to influence are morphology (Lardiere, 1998b; Slabakova, 2008), as well as aspects that lie outside core aspects of syntax, which are inherently unstable, because of their dependence on the context (Montrul, 2004; Serratrice et al., 2004). This flexibility of licensing can in fact be found in Spanish, where instances of perspective shift emerge in the adult acceptability data though not in production. However, they do emerge in the children's production data. Thus, English is not influencing the language, forcing perspectival assimilation where it is otherwise not licensed, but rather interacting with pre-existing licensing conditions.

The fact that this effect lies at the interface of semantics and pragmatics is precisely what is most striking about it. It is not until the restrictions encoded in the Spanish entry are learned and other more precise verbs, such as *salir* or *aparecer* take preference over *venir* in production can *block* this usage of *venir* as more specific or optimal alternatives, thereby pruning it away. That some other aspects of the bilingual children's linguistic competence remain largely unaffected by this cross-linguistic influence is not surprising; dissociations between syntax and morphology have been documented across a number of studies (Lardiere, 1998a,b), and it is fairly well-accepted by now that lexical and morphosyntactic knowledge are stored separately, albeit in L2 adults (Ullman, 2001). Moreover, as we noted in the introduction, areas, such as lexical semantics and areas that depend on context are more susceptible to influence from the dominant language (Montrul, 2004).

The present research, in which a production study of deictic verbs in narratives by heritage bilingual children and monolingual child and adult populations is complemented with a judgment Spain study from Spanish speaking adults, affords us a unique opportunity to observe more precisely how some patterns of perspective with limited but existing acceptance among adults may show up in child production data and become more prevalent in contexts of language contact. Importantly, it has revealed a less strict set of constraints on perspective shift with *venir* in Spanish. Uncovering this would not have been possible by focusing solely on production or on one population of speakers alone. The fact that there were dialectal differences between the groups of monolingual and bilingual adults in the judgment study points to a possible ongoing change occurring in Spanish that future research should address. Future research is needed to further probe the acceptability and production of these non-speaker-oriented instances of *venir* in order to refine the discourse licensing conditions of *venir* in Spanish.

## Data Availability Statement

The original contributions presented in the study are included in the article/supplementary material, further inquiries can be directed to the corresponding author/s.

## Ethics Statement

The studies involving human participants were reviewed and approved by Rutgers ORSP (IRB Protocol 12-042Mc). Written informed consent to participate in this study was provided by the participants' legal guardian/next of kin.

## Author Contributions

All authors contributed to the selection of the corpora, analysis of the corpus search data, design of the acceptability experiments, analysis of the acceptability data, theoretical analysis and proposal, and the writing and editing of the manuscript.

## Conflict of Interest

The authors declare that the research was conducted in the absence of any commercial or financial relationships that could be construed as a potential conflict of interest.

## Footnotes

^1^Verde (2014) examined judgment data on deictic verbs of motion used to describe video clips from English monolingual adult speakers and Spanish-English bilingualsfound that the bilinguals did not differ from the monolinguals.

^2^Note that in this particular case, the bilingual participants' dominant language (English) also happens to be the language with the more flexible parameters for deictic verbs. This pattern certainly isn't necessarily the case for all bilinguals, so the question arises, what might happen if the situation were reversed.

^3^We highlight these grammatical features in particular, because they have been shown to be common indicators of cross-linguistic influence from English in Spanish heritage speakers (Montrul, 2016).

^4^Spanish is a language that requires masculine or feminine gender agreement between articles, adjectives and nouns. English does not have gender agreement of any kind (Camacho, 2013).

^5^One filler item was omitted in the calculation for the US group. This item with number disagreement “dos maleta” (two suitcase) was accepted at a rate of 88% which may be due to the ubiquity of /s/ aspiration in Caribbean Spanish (Lynch, 2009).
