# Supplementary material for: Perspective-Taking With Deictic Motion Verbs in Spanish: What We Learn About Semantics and the Lexicon From Heritage Child Speakers and Adults
Source: Front Psychol. 2021 Mar 31;12:611228. doi: 10.3389/fpsyg.2021.611228 (PMC8044305; doi:10.3389/fpsyg.2021.611228)
Supplement: Supplementary file 1 [file Data_Sheet_1.pdf]

Appendix A: Examples of verbal productions (including *venir*) across the five groups of Spanish speakers for each scene

(a) **Frog**
llamar (call), *llamar, pero [verbos varios]* (call, but [other verb]), *llamar, pero no venir* (call, but not come)

SPANISH	Spanish adults		8/9-year-olds Spain		6-year-olds Mexico		12-year-olds Mexico		**Spanish heritage**	
llamar	15	83%	29	57%	4	100%	19	90%	107	87%
llamar, pero [verbos varios]	3	17%	4	8%	0	0%	0	0%	11	9%
llamar, pero no venir	0	0%	1	2%	0	0%	2	10%	5	4%
Total Vs	18	100%	51	100%	4	100%	21	100%	123	100%

(b) **Mole**
aparecer (appear), *encontrar* (find), *morder*/*picar* (bite/sting), *salir* (go out), *otros* varios (other verbs)

SPANISH	Spanish adults		8/9-year-olds Spain		6-year-olds Mexico		12-year-olds Mexico		**Spanish heritage**	
aparecer	1	10%	0	0%	1	11%	0	0%	0	0%
encontrar	1	10%	2	7%	1	11%	0	0%	4	7%
morder/picar	0	0%	4	13%	0	0%	0	0%	13	23%
salir	6	60%	20	67%	6	67%	8	80%	31	55%
otros varios	3	30%	4	13%	1	11%	2	20%	8	14%
venir	0	0%	0	0%	0	0%	0	0%	0	0%
Total Vs	10	100%	30	100%	9	100%	10	100%	56	100%

(c) **Bees**
coger (pick up), *correr* (run), *corretear* (surround), *(per)seguir* (follow, chase), *salir* (go out), *ir* (go), *otros varios* (other verbs)

SPANISH	Spanish adults		8/9-year-olds Spain		6-year-olds Mexico		12-year-olds Mexico		**Spanish heritage**	
coger	0	0%	0	0%	0	0%	0	0%	4	6%
correr	0	0%	0	0%	0	0%	0	0%	5	7%
corretear	0	0%	0	0%	0	0%	3	30%	0	0%
(per)seguir	0	0%	6	20%	4	42%	5	50%	18	25%
salir	7	70%	16	53%	5	42%	1	10%	17	24%
ir	0	0%	5	17%	0	0%	0	0%	15	21%
otros varios	3	30%	1	3%	2	17%	1	10%	11	16%

venir	0	0%	1	3%	0	0%	0	0%	0	0%
Total Vs	10	100%	30	100%	12	100%	10	100%	70	100%

(d) **Owl**

aparecer (appear), *asustar* (frighten), *hacer caer* (make fall), *ver* (see), *haber* (be/take place), *salir* (go out), *otros* varios (other verbs)

SPANISH	Spanish adults		8/9-year-olds Spain		6-year-olds Mexico		12-year-olds Mexico		**Spanish heritage**	
aparecer	1	17%	1	3%	0	0%	0	0%	0	0%
asustar	0	0%	3	10%	0	0%	1	10%	2	5%
hacer caer	0	0%	0	0%	0	0%	0	0%	3	5%
ver	0	0%	0	0%	0	0%	0	0%	4	7%
haber	0	0%	3	10%	0	0%	1	10%	5	9%
salir	5	83%	19	63%	6	67%	6	60%	29	50%
otros varios	0	0%	4	13%	3	33%	2	20%	12	21%
venir	0	0%	0	0%	0	0%	0	0%	2	3%
Total Vs	6	100%	30	100%	9	100%	10	100%	58	100%

(e) **Deer**

aparecer (appear), *(re)coger* (pick up), *encontrar* (find), *ser/haber* (be/take place), *sostener* (hold), *salir* go out), *otros* varios (other verbs)

SPANISH	Spanish adults		8/9-year-olds Spain		6-year-olds Mexico		12-year-olds Mexico		**Spanish heritage**	
aparecer	0	0%	1	3%	1	10%	0	0%	0	0%
(re)coger	0	0%	3	30%	0	0%	0	0%	10	13%
encontrar	1	11%	2	7%	1	10%	0	0%	7	9%
ser/haber	0	0%	11	37%	0	0%	8	80%	17	22%
sostener	0	0%	4	13%	2	20%	0	0%	18	23%
salir	5	67%	19	63%	1	10%	0	0%	9	11%
otros varios	2	22%	2	7%	5	50%	2	20%	15	19%
venir	0	0%	0	0%	0	0%	0	0%	3	4%
Total Vs	9	100%	30	100%	10	100%	10	100%	79	100%

(f) **Frog family**
estar (be), *haber* (be/take place), *aparecer* (appear), *llegar* (arrive), *ir* (go), *salir* (go out)

SPANISH	Spanish adults		8/9-year-olds Spain		6-year-olds Mexico		12-year-olds Mexico		**Spanish heritage**	
estar	2	100%	5	36%	0	0%	0	0%	17	57%
haber	0	0%	1	7%	0	0%	1	33%	5	17%
aparecer	0	0%	2	14%	0	0%	0	0%	0	0%
llegar	0	0%	0	0%	0	0%	1	33%	0	0%
ir	0	0%	0	0%	0	0%	0	0%	2	7%
salir	0	0%	6	43%	2	100%	1	33%	4	13%
venir	0	0%	2	14%	0	0%	0	0%	2	7%
Total Vs	2	100%	14	100%	2	100%	3	100%	30	100%

Appendix B: Productions from corpus study

Spanish heritage speakers

English

(1) He called his name but nobody **came** (SP/ENG HER-10/11: 22132099)

(2) This deer **came** along and picked up the boy (SP/ENG HER-10/11: 22232065)

(3) A big owl **came** and scared him (SP/ENG HER-10/11: 11232167)

(4) A bird **came** and tried to **attack** him (SP/ENG HER-10/11: 22232410)

Spanish

(5) la llamó y la llamó y no **vino** (SP/ENG HER-10/11: 22132056)

it he.called and it he.called and [the frog] NEG come

'He called and he called, but the frog didn't come.'

(6) y llamaron rana, rana, pero no **vino** (SP/ENG HER-10/11: 11132198)

and they.called frog, frog, but NEG come

'And they called 'frog, frog' but the frog didn't come.'

(7) llamaron son nombre pero nadie **vino** (SP/ENG HER-10/11: 22132099)

they.called his name but no one came

'They called his name but no one came.'

(8) cuando **vino** un reindeer (SP/ENG HER-10/11: 22132056)

when came a reindeer

'When a reindeer came along.'

(9) pero **vino** el reindeer (SP/ENG HER-10/11: 11131281)

but came the reindeer

'But the reindeer came.'

English monolingual speakers

(10) A mole **came** and **pinched** him on the nose (ENG-adult: 31332118)

(11) Then a bird **came** (ENG-adult: 31331470)

(12) Then a deer **came** (ENG-adult: 31332236)

Spanish monolingual speakers

(13) y de repente **salió** un topo (MEX-12: 1204)

and suddenly went.out a. mole

'And suddenly a mole comes out.'

(14) después **salió** una lechuza (MEX-12: 09b)

after went.out an owl

'Then an owl came out.'

(15) y del agujero **sale** un buho (MEX-12: 09c)

and from.the hole goes.out an owl

'And an owl comes out of the hole.'

(16) **aparece** un buho (SP-8/9: yb0801)

there.appears an owl

'An owl appears'

(17) entonces **aparece** un buho (SP-adult: 20b)

then there.appears an owl

'Then an owl appears'

(18) el ciervo **sale** corriendo (MEX-12: 09f)

the deer goes.out running

'The deer comes running out,'

(19) y esas ramas no eran ramas sino **era** un venado (MEX-12: 1209)

and those branches NEG were branches instead was a deer

'And those branches were not branches. Rather, they were a deer'

(20) y las abejas lo **están persiguiendo** (MEX-6: 602)

and the bees him are chasing

'And the bees are chasing him'

(21) luego las abejas empezaron **a perseguir** al perro (MEX-12: 1207)

then the bees started to chase to.the dog

'Then the bees started chasing the dog'

Appendix C: Test and filler items in the Acceptability Study

Motion away from the protagonist using *ir*

(22) Jaime y Sara son compañeros de trabajo.

Jaime and Sara work together.

Esta mañana, Jaime **fue** a la oficina de Sara para verla.

This morning, Jaime **went** to Sara's office to see her.

(23) David y Alex son amigos.

David and Alex are friends.

Ayer, David **fue** al cine con Alex.

Yesterday, David **went** to the movies with Alex.

(24) Teresa es futbolista.

Teresa is a soccer player.

La semana pasada su equipo **fue** a California para jugar un partido.

Last week, her team **went** to California to play a game.

(25) Hace 5 años, Charlie estaba de viaje en Hawaii.

Mientras estaba allí, **fue** a ver un volcán.

Five years ago, Charlie was on a trip in Hawaii.

While he was there, he **went** to see a volcano.

(26) Violeta era profesora.

Un día, **fue** al trabajo y sus estudiantes la soprendieron con un regalo muy bonito.

Violeta was a teacher.

One day, she **went** to work and her students surprised her with a beautiful gift.

Motion away from the protagonist using *venir*

(27) Jorge estaba de paseo por el campo.

De repente, **vino** a un árbol enorme y majestuoso.

Jorge was walking through the countryside.

Suddenly, he **came** to an enormous and majestic tree.

(28) Hace tres años Martín se mudó para la ciudad.

Este verano, **vino** a la casa de sus abuelos en el campo por 2 semanas.

Three years ago, Martin moved to the city.

This summer, he **came** to his grandparents' house in the country for 2 weeks.

(29) El otro día, Lola salió con sus amigas.

Cuando **vino** al restaurante, estaba entusiasmada.

The other day, Lola went out with her friends.

When she **came** to the restaurant, she was excited.

(30) Roberto estaba en una excursión en la selva.

De repente, **vino** a un río con cocodrilos.

Roberto was on an adventure in the jungle.

Suddenly, he **came** to a river with crocodiles.

(31) Carlos estaba nadando en el mar.

Después de un rato, **vino** a unas rocas y descansó.

Carlos was swimming in the sea.

After a while, he **came** to some rocks and rested.

Motion toward a hearer in dialogue using *ir*

(32) Lucía está en Sevilla y Fátima está en Londres. Están hablando por teléfono cuando Lucía le dice, “**Voy** a Sevilla mañana para verte.”

Lucía was in Sevilla and Fátima was in London. They are talking on the phone when Lucía says, “I’m **going** to Sevilla tomorrow to see you.”

(33) Alicia tiene un regalo para su abuela. Esta mañana la llamó y le dijo,

“**Voy** a tu casa luego para darte el regalo.”

Alicia has a gift for her grandmother. This morning she called her and said,

“I’m **going** to your house later to give you a gift.”

(34) Al llegar del trabajo, el padre de Oscar vio que no había terminado su tarea. Lo llamó desde la sala y Oscar le contestó,

“¡Ya **voy**, papá!”

Upon arriving from work, Oscar's dad saw that he hadn't finished his homework. He called him from the living room and Oscar responded,

"I'm **going**, dad!"

(35) Lucas se cayó de su bicicleta y llamó a su hermano que iba delante. Su hermano se dio la vuelta y chilló,

“¡Ya **voy** hermano!”

Lucas fell off his bike and called for his big brother who was in front of him. His brother turned around and shouted,

“I'm **going**, brother!”

(36) Adriana llamó a su amiga por teléfono para pedirle ayuda con un proyecto de la escuela. Su amiga le dijo,

“**Voy** para tu casa ahora mismo para ayudarte.”

Adriana called her friend on the phone to ask for help with a school project. Her friend said, “I'm **going** to your house right not to help you.”

Motion toward a hearer in dialogue using *venir*

(37) Al llegar a casa, la mamá de Angelica se encontró con un desorden en la sala. Molesta, llamó a Angelica y ella le respondió:

“¡Ya **vengo**, mamá!”

Upon arriving home, Angelica’s mom found a disaster in the living room. Angry, she called Angelica and she responded:

“I’m **coming**, mom!”

(38) Samuel vio un vídeo buenísimo en Instagram y llamó a Diego para enseñárselo. Diego le contestó: “¡Ya **vengo**, Samuel!”

Diego and Samuel are housemates. Samuel saw a great video on Instagram and called Diego to show him. Diego answered him: “I’m **coming**, Samuel!”

(39) Laura encontró un insecto raro en el jardín y quería enseñárselo a su papá. Lo llamó y el papá respondió: “¡Ya **vengo**, Laura!”

lLaura found a strange insect in the garden and wanted to show it to her dad. She called him and her dad answered: “I’m **coming**, Laura!”

(40) Emilio y Enrique están hablando por teléfono. Emilio le cuenta que tiene un videojuego nuevo. Enrique le contesta,

"**Vengo** a tu casa ahora mismo para jugarlo contigo."

Emilio and Enrique are talking on the phone. Emilio says he has a new videogame. Enrique replies, "I'm **coming** over right now to play with you."

(41) Esta noche hay una gran fiesta en la casa de Darío. Raúl lo llama para confirmar su asistencia y le dice, “¡**Vengo** a tu fiesta esta noche!”

Tonight there is a big party at Dario's house. Raul calls him to confirm he is attending and says, “I'm **coming** to your party tonight!”

Motion toward the protagonist using *venir* with a null subject

(42) Alejandro estaba paseando a su perro. De repente, el perro se soltó de la correa para perseguir a una ardilla.

Alejandro lo llamó, pero no **vino**.

Alejandro was walking his dog when he got loose chasing a squirrel.

Alejandro called him, but he didn’t **come**.

(43) Hoy fue el papá de Julia a recogerla de la guardería.

La llamó desde la puerta de la clase, pero Julia estaba jugando y no **vino**.

Today Julia’s dad went to pick her up from school.

He called her from the classroom door, but Julia was playing and didn’t **come**.

(44) Alvaro vive en el campo. Un día, no pudo encontrar a su gato.

Lo llamó por toda la casa, pero no **vino**.

Alvaro lives in the countryside. One day he couldn’t find his cat.

He called all around the house, but she didn’t **come**.

(45) María tenía un pajarito que se escapó.

Lo llamó y lo llamó pero no **vino**.

Maria had a pet bird that escaped.

She called it and called it, but it didn't **come**.

(46) Nelson estaba jugando en el jardín.

Su madre lo llamó para que entrara a cenar, pero no le hizo caso y no **vino**.

Nelson was playing in the garden.

His mother called him to eat dinner, but he ignored her and didn't **come**.

Motion toward the protagonist using *venir* with VS word order

(47) Laila soñó que estaba en el país de las maravillas y que había animales fantásticos por todas partes.

Vino un unicornio y le lamió la mano.

Laila dreamed that she was in Wonderland and there were fantastical animals everywhere.

A unicorn **came** and licked her hand.

(48) Valentina era una cantante muy buena.

Vino mucha gente a su primer concierto.

Valentina was a very good singer.

A lot of people **came** to her first concert.

(49) Las joyas de la reina eran preciosas.

Un día, **vino** un rey de otro país para ver las joyas y decidió comprarlas.

The queen's jewels were beautiful.

One day, **came** another king to see the jewels and decided to buy them.

(50) Correr maratones es muy dificil, pero Nina es una campeona.

Un día, **vino** una niña de 7 años para preguntarle si la ayudaría a entrenar.

Running marathons is hard, but Nina is a champion.

One day, a 7 year old girl **came** to ask her if she would train her.

(51) Juan vivía en una ciudad muy grande.

Un día salió a la calle y **vino** un mendigo a pedirle dinero.

Juan lived in a big city.

One day when he went out and a beggar **came** to ask him for money.

Motion toward the protagonist using *venir* with SV word order

(52) Ayer Stephanie hizo un pastel de chocolate divino y llamó a su amiga Cristina para que lo probara. Cristina **vino** a la cocina inmediatamente.

Yesterday Stephanie baked a delicious chocolate cake and she called her friend Cristina to try it. Cristina **came** to the kitchen right away.

(53) Daniel ha estado en el hospital durante una semana después de haber tenido un accidente.

Hoy, su amigo Antonio **vino** a visitarlo al hospital.

Daniel has been in the hospital for a week after an accident.

Today, Antonio **came** to visit him in the hospital.

(54) Una vez, un niño viajó al Polo Norte para buscar a Papá Noel.

Mientras caminaba por la nieve, un reno **vino** al niño y lo miró a los ojos.

One time, a child traveled to the North Pole to look for Santa Claus.

While he was walking through the snow, a reindeer **came** to the boy and looked him in the eyes.

(55) Raquel y Andrea son amigas de la escuela.

Hoy, Andrea **vino** a la casa de Raquel para estudiar.

Raquel and Andrea are school friends.

Today, Andrea **came** to Raquel's house to study.

(56) Hace 1 año, Bernardo se hizo famoso.

De repente, mucha gente **vino** a su casa para intentar verlo.

One year ago, Bernardo became famous.

Suddenly, lots of people **came** to his house to try to see him.

Fillers

(57) Petra le regaló unos zapatos a Clara por su cumpleaños.

Emocionada, Clara se puso ***el zapatos nuevos**.

Petra gave Clara new shoes for her birthday.

Excited, Clara put on **the new shoes**.

(58) Rodrigo se fue de viaje a Sudáfrica.

Se llevó ***dos maleta**.

Rodrigo went on a trip to South Africa.

He took **two suitcase**.

(59) Irene preparó sus materiales para la escuela. Tenía tres libros dentro de la mochila.

Solo tenía **un lápices**.

Irene prepared her school materials. She had three books in her backpack.

She only had **one pencils**.

(60) Zoe fue a la tienda a comprar algunas cosas que quería.

Le encantan sus ***nuevos cadenas de oro**.

Zoe went to the store to buy some new things she wanted.

She loves her **new gold chains**.
